# Setting the Stage: The Role of Navigation in Dementia Care

**DOI:** 10.1002/alz70858_110738

**Published:** 2025-12-26

**Authors:** Katherine Evans

**Affiliations:** ^1^ Alzheimer's Association, Chicago, IL, USA

## Abstract

This session will provide an introduction to dementia care coordination and navigation, including the Alzheimer's Association's role in these initiatives. Additionally, an overview of the GUIDE Model, including the program's associated goals and potential impact on dementia care will be discussed.

